# Comparison of synthetic aperture architectures for miniaturised ultrasound imaging front-ends

**DOI:** 10.1186/s12938-018-0512-6

**Published:** 2018-06-18

**Authors:** Graham Peyton, Martyn G. Boutelle, Emmanuel M. Drakakis

**Affiliations:** Department of Bioengineering, Imperial College London, South Kensington Campus, London, SW7 2AZ UK

**Keywords:** Compressed sensing, Portable ultrasound, Quadrature sampling, Synthetic aperture imaging

## Abstract

**Background:**

Point of care ultrasonography has been the focus of extensive research over the past few decades. Miniaturised, wireless systems have been envisaged for new application areas, such as capsule endoscopy, implantable ultrasound and wearable ultrasound. The hardware constraints of such small-scale systems are severe, and tradeoffs between power consumption, size, data bandwidth and cost must be carefully balanced.

**Methods:**

In this work, two receiver architectures are proposed and compared to address these challenges. Both architectures uniquely combine low-rate sampling with synthetic aperture beamforming to reduce the data bandwidth and system complexity. The first architecture involves the use of quadrature sampling to minimise the signal bandwidth and computational load. Synthetic aperture beamforming (SAB) is carried out using a single-channel, pipelined protocol suitable for implementation on an FPGA/ASIC. The second architecture employs compressive sensing within the finite rate of innovation framework to further reduce the bandwidth. Low-rate signals are transmitted to a computational back-end (computer), which sequentially reconstructs each signal and carries out beamforming.

**Results:**

Both architectures were tested using a custom hardware front-end and synthetic aperture database to yield B-mode images. The normalised root-mean-squared-error between the quadrature SAB image and the RF reference image was $$13\%$$ while the compressive SAB error was $$22\%$$ for the same degree of spatial compounding. The sampling rate is reduced by a factor of 2 (quadrature SAB) and 4.7 (compressive SAB), compared to the RF sampling rate. The quadrature method is implemented on FPGA, with a total power consumption of $$4.1 $$ mW, which is comparable to state-of-the-art hardware topologies, but with significantly reduced circuit area.

**Conclusions:**

Through a novel combination of SAB and low-rate sampling techniques, the proposed architectures achieve a significant reduction in data transmission rate, system complexity and digital/analogue circuit area. This allows for aggressive miniaturisation of the imaging front-end in portable imaging applications.

## Background

Recent years have seen significant advances in the development of highly portable ultrasound imaging systems providing real-time diagnostic information. These developments have largely been fueled through parallel advances in integrated electronics, micromachined transducers and signal processing methods. Field-programmable gate arrays (FPGAs) and application-specific integrated circuits (ASICs) provide adaptive parallelism, high performance per milliwatt, and a smaller form factor. Much work has also focused on integrating electronic front-ends with novel capacitive/piezoelectric micromachined ultrasound transducers (CMUTs/PMUTs) for tethered applications such as 3D intravascular or endoscopic imaging [1, 2]. This has led to an ever increasing drive to further miniaturise point-of-care ultrasound devices. However, many challenges still inhibit the development of further miniaturised, wireless systems for novel applications such as ultrasonic capsule endoscopy [3] and wearable imaging [4]. This is primarily due to the severe power and circuit area constraints affecting both analogue and digital components of the system. Cost has also been a major limitation in low-resource clinical settings, and while some more affordable commercial devices like the GE VScan have been devised, there remains scope for further reductions in system complexity and cost. The critical challenge is maintaining sufficient image quality for diagnostic purposes while reducing size, system complexity and cost. These considerations form the basis of this investigation, where two ultrasound receiver architectures are proposed in an attempt to optimise these tradeoffs.

A great diversity of efficient hardware-level beamforming strategies in the analogue and digital domains have been proposed in literature. In analogue beamforming, signals from each transducer element are delayed with analogue delay lines, and then summed and digitised [5–7]. However, the number of analogue delay cells increases quadratically with the number of channels, resulting in excessive complexity, power consumption and pulse distortion for a practical array. Digital RF beamforming systems have the advantage of better delay accuracy, provided that the clock frequency is high enough. If conventional phased array imaging is used with many parallel receive channels, the price is increased analogue-to-digital converter (ADC) power consumption and data bandwidth. Kim et al. attempt to alleviate this problem by multiplexing many channels through a single ADC [8]. They propose a CMOS ultrasound transceiver chip that is closely coupled to a $50$ MHz PMUT transducer with 16 elements. However, a high clock frequency ($250$ MHz) is required, and the overall power consumption ($270$ mW) exceeds the constraints of a highly portable, wireless application. Furthermore, receive beamforming is not carried out on-chip. In [9], a state-of-the-art, 32-channel, point-of-care system-on-a-chip (SOC) is demonstrated, featuring full transmit and dynamic receive beamformer modules and colour Doppler processors. However, the SOC consumes $1.2$ W and occupies $27\times 27$ mm^2^ (for $32-64$ channels).

In-phase/quadrature (I/Q) beamforming techniques have also been proposed to reduce hardware complexity. The direct sampled I/Q beamforming method in [10] employs second-order sampling to obtain I/Q components directly from RF signals. Digital focusing is then implemented via phase rotation of the I/Q data. This considerably reduces the hardware requirements, making the beamformer small and inexpensive. In [11] a similar phase-error-free quadrature sampling technique is used, where I/Q components are obtained by mixing with a reference signal. I/Q data are then sampled and focused using synthetic aperture beamforming (SAB). While SAB is particularly useful in small scale systems in which hardware simplicity is mandatory [11, 12], it does lead to lower SNR and unwanted grating lobes. However, numerous techniques have been proposed to assist with minimising these grating lobes and increasing the SNR [12].

Compressive sensing (CS) is another viable method of improving power efficiency by decreasing the sampling rate. In [13], Vetterli et al. proposed a sampling paradigm for certain classes of parametric signals with a finite rate of innovation (FRI). This work is extended by Eldar et al. [14] into a unified framework termed *Xampling* and applied it to ultrasound imaging in software [15]. The result is an eightfold reduction in sampling frequency from the original RF rate, with comparable image quality.

In this work, the synthetic aperture beamforming (SAB) is applied as a means of reducing system complexity [11, 12]. A novel SAB method is employed which combines aspects of the traditional synthetic aperture focusing technique (SAFT), and synthetic receive aperture (SRA) beamforming, where the receive aperture is split into multiple sub-apertures that are multiplexed in time. In this system, transmission is carried out *n* times for all receive elements, and reflected signals are multiplexed through a single receive channel, which significantly reduces system complexity and size. Spatial compounding across multiple transmit positions increases the SNR. Although only a single channel is used, the entire system is scalable to any number of channels, depending on what frame rate is required.

Furthermore, SAB is uniquely combined with two different sampling techniques: quadrature/baseband sampling and compressive sensing within the FRI framework. This approach enables unprecedented miniaturization through a reduction system complexity and data bandwidth. Both architectures are tested in hardware and compared in terms of their utility in highly portable applications. The architectures do not challenge existing solutions such as [9], which focus on portable applications with high image quality ($\sim 128$ channels) and high frame rates ($\sim 30$ Hz). Instead, frame rate and image quality are carefully traded against hardware complexity, cost and power consumption in order to enable further miniaturisation for small-scale applications such as capsule endoscopy (which typically requires a frame rate of 2–4 Hz). Importantly, the parameters of both architectures may be tuned to balance these tradeoffs for the given application.

## Methods

### Architecture 1: compressive sensing with the FRI framework

The first architecture employs a sampling paradigm originally proposed by Vetterli et al. [13] for certain classes of parametric signals. Parametric signals with *k* parameters may be sampled and reconstructed using only 2*k* parameters. These signals have a *finite rate of innovation *(FRI) and appear in many applications such as biomedical imaging and radar. The sampling scheme in [13] was applied to periodic and finite streams of FRI signals such as Diracs impulses, nonuniform splines, and piecewise polynomials. An appropriate sample kernel (sinc, Guassian, sum of sincs, etc. [15]) is applied to extract a set of Fourier coefficients which are then used to obtain an annihilating filter. The locations and amplitudes of the pulses are finally determined. A brief review of the method in [13] is provided below.

Consider an FRI signal *x*(*t*) (e.g., ultrasound A-mode signal) comprising a finite stream of pulses with pulse shape *p*(*t*), amplitudes $\left\{ c_{k}\right\} _{k=0}^{K-1}$ and time locations $\left\{ t_{k}\right\} _{k=0}^{K-1}$:

1 $$\begin{aligned} x(t)= & {} \sum _{k=0}^{K-1}c_{k}p(t-t_{k}) \end{aligned}$$

The sample values are obtained by filtering the signal with a sampling kernel. A sinc kernel is defined as ${h_{B}\left( t\right) =B \mathrm{sinc}\left( Bt\right) }$, with bandwidth $B=1/T$. The convolution product is:

2 $$\begin{aligned} y_{n}=  \left\langle h_{B}\left( t-nT\right) ,x\left( t\right) \right\rangle \quad n=0,\,\ldots ,\,N-1 \end{aligned}$$

This is equivalent to:

3 $$\begin{aligned} y_{n}&=  \sum _{k=0}^{K-1}c_{k}B\,\mathrm{sinc}\left( \frac{t_{k}}{T}-n\right) \end{aligned}$$

4 $$\begin{aligned}&=  \left( -1\right) ^{n}\sum _{k=0}^{K-1}\frac{c_{k}B\,\mathrm{sin}\left( \frac{\pi t_{k}}{T}\right) }{\pi \left( \frac{t_{k}}{T}-n\right) }\end{aligned}$$

5 $$\begin{aligned} &\iff \left( -1\right) ^{n}y_{n}= & {} \frac{1}{\pi }\sum _{k=0}^{K-1}c_{k}B\,\mathrm{sinc}\left( \frac{\pi t_{k}}{T}\right) \frac{1}{\left( \frac{t_{k}}{T}-n\right) } \end{aligned}$$

Since the signal has *K* degrees of freedom, we require $N\ge 2K$ samples to sufficiently recover the signal. The reconstruction method requires two systems of linear equations—one for the locations of the Gaussian pulses involving a matrix *V*, and one for the weights of the pulses involving a matrix *A*. Define a Lagrange polynomial $L_{k}\left( u\right) =\left( P\left( u\right) /\left( u-t_{k}/T\right) \right)$ of degree $K-1$, where $P\left( u\right) =\prod _{k=0}^{K-1}\left( u-t_{k}/T\right) .$ Multiplying both sides of (5) by $P\left( n\right)$ yields an expression in terms of the interpolating polynomials:

6 $$\begin{aligned} \underbrace{\left( -1\right) ^{n+1}P\left( n\right) y_{n}}_{\mathbf {Y}_{n}}= & {} \sum _{k=0}^{K-1}c_{k}\underbrace{B\,\mathrm{sin}\left( \frac{\pi t_{k}}{T}\right) \,\frac{L_{k}\left( n\right) }{\pi }}_{\left[ \mathbf {A}\right] _{nk}}\end{aligned}$$

7 $$\begin{aligned} \Longleftrightarrow \mathbf {Y}=  \mathbf {A}.\mathbf {c} \end{aligned}$$

To find the *K* locations $t_{k}$ (i.e. the time delays of the pulses), we begin by deriving an annihilating equation to find the roots of $P\left( u\right)$. Now, since the right hand side of (6) is a polynomial of degree $K-1$ in the variable *n*, if we apply *K* finite differences, the left hand side will become zero, i.e., $\triangle ^{K}\left( \left( -1\right) ^{n}P\left( n\right) y_{n}\right) =0,\quad n=K,\,\ldots ,\,N-1$. Letting $P\left( u\right) =\sum _{k}p_{k}u^{k}$ leads to an annihilating filter equation equal to:

8 $$\begin{aligned} \sum _{k=0}^{K}p_{k}\underbrace{\triangle ^{K}\left( \left( -1\right) ^{n}n^{k}y_{n}\right) }_{[\mathbf {V}]_{nk}}=  0\end{aligned}$$

9 $$\begin{aligned} \Longleftrightarrow \mathbf {V}.\mathbf {p}=  0 \end{aligned}$$

where $\mathbf {V}$ is an $\left( N-K\right) \times \left( K+1\right)$ matrix. The system admits a solution when ${\mathrm{Rank}\left( \mathbf {V}\right) }\le K$ and $N\ge 2K$. Thus, (8) may be used to find the $K+1$ unknowns $p_{k}$, which leads to *K* locations $t_{k}$ as these are the roots of $P\left( u\right)$. Once the locations have been determined, the weights of the Gaussian pulses $c_{k}$ may be found by solving the system in (7) for $n=0,\,\ldots ,\,K-1$. The system has no solution if ${\mathrm{Rank}\left( \mathbf {A}\right) }=K$, where ${\mathbf {A}\in \mathrm{I\!R}^{K\times K}}$ is defined by (6). A more detailed discussion of the annihilating filter method is provided in [13]. Theoretically, the result does not depend on the sampling period *T*. However, $\mathbf {V}$ may be poorly conditioned if *T* is not chosen appropriately. As simulation results show below, oversampling yields an increase in the SNR of the reconstructed result.

It is also important to note that the sinc kernel described above has infinite time support and is non-causal. In the frequency domain, it is represented by an ideal lowpass filter with an infinite rolloff. Practically, the sinc kernel may be approximated in hardware by means of an high order analogue lowpass filter. Simulations below demonstrate the performance of multiple filter types and orders, and a comparison is made to other kernel types suggested in [15].

#### System overview

**Fig. 1:**
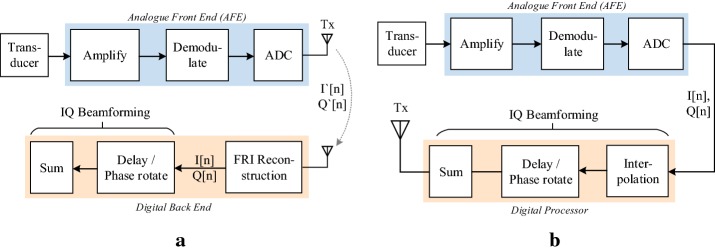
I/Q beamforming architectures. **a** Compressive SAB: low-rate samples are transmitted to a computational back-end for reconstruction and beamforming. **b** Quadrature SAB: synthetic aperture beamforming is carried out digitally in the baseband to form a 2D image, which may then be transmitted to a display device

The proposed compressive synthetic aperture beamforming (SAB) architecture is illustrated in Fig. 1a. The analogue front-end (AFE) amplifies and demodulates the RF signal into I and Q components. This is achieved by mixing the RF waveform with reference signals centered at the carrier frequency. The assumption is made that both the I and Q signals satisfy the FRI criterion, i.e., they both have finite rates of innovation. These signals are thus filtered and bandlimited *below* the original I/Q bandwidth. This is carried out in the analogue domain in order to reduce the sampling frequency and thus the data bandwidth. This leads to a significant power saving, as the power budget is predominated by the power consumption of the ADC and wireless transceiver. By compressing the signal in the analogue domain, the computational burden is shifted to the digital back end (computer), which carries out reconstruction of the I/Q signals and finally baseband beamforming. Power is not a critical constraint here as it is in the second architecture (quadrature SAB), where beamforming is carried out on FPGA.

**Fig. 2:**
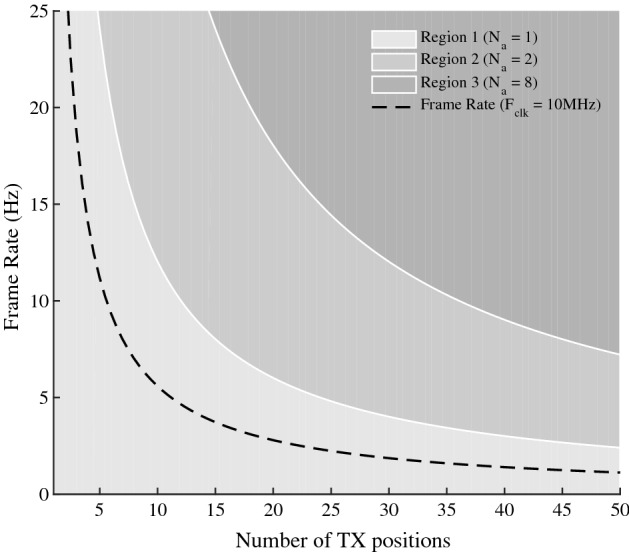
Frame rate versus the number of transmit positions $i_{max}$

**Fig. 3:**
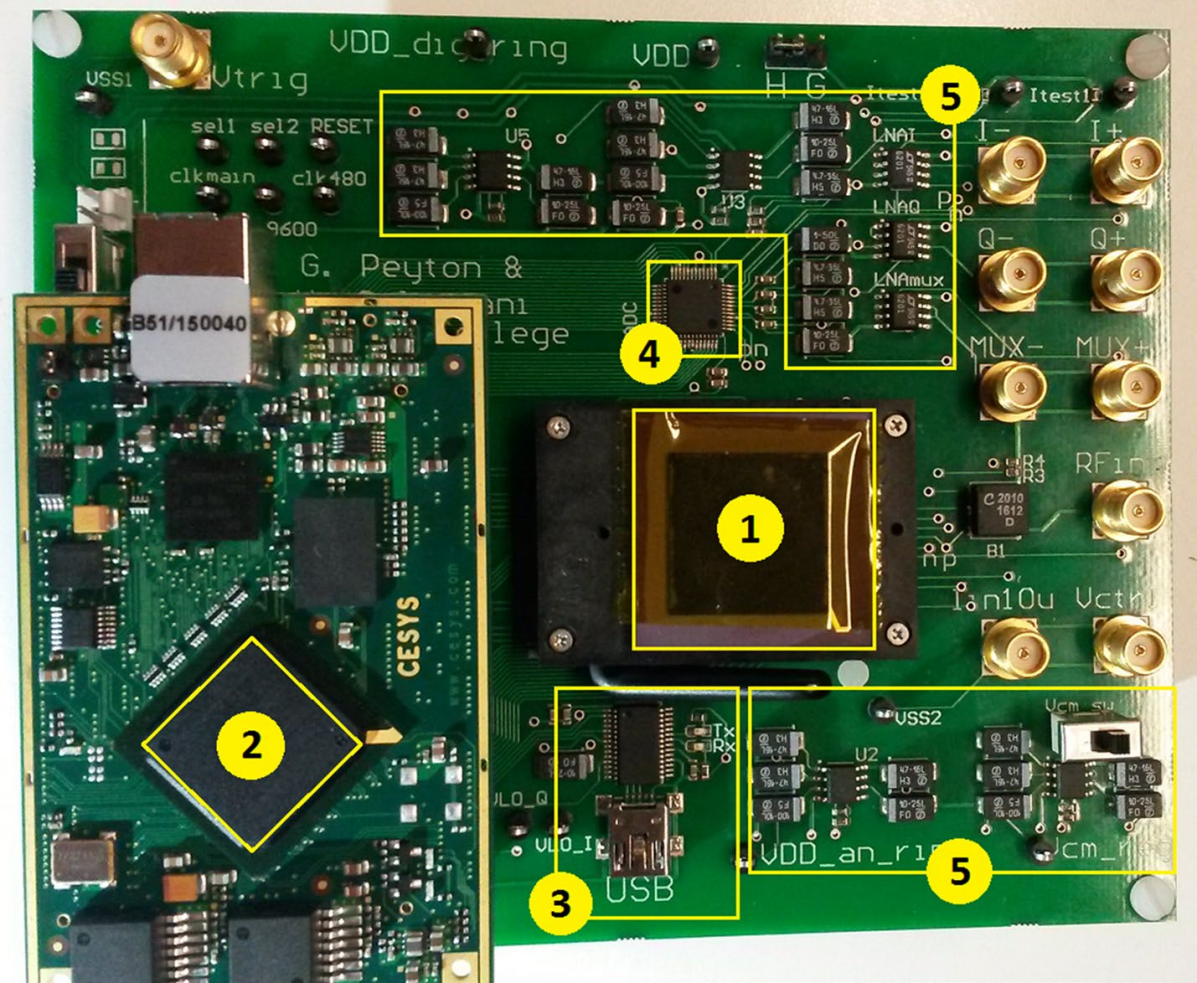
Photograph of the PCB used for testing the AFE and beamforming algorithm on FPGA. (1) AFE (2) Spartan-6 on EFM-02 development board (3) UART FT232 chip USB connector (4) ADC10D020 Dual-Channel ADC. (5) ADM7155 voltage regulators

**Fig. 4:**
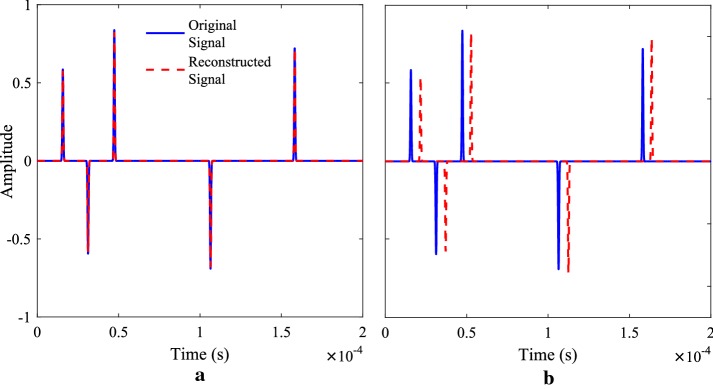
Simulating a noiseless stream of random Dirac pulses ($L=5,$ $F=4$). Original versus reconstructed signals are compared for (**a**) a sinc filter, **b** 4th order LPF

**Fig. 5:**
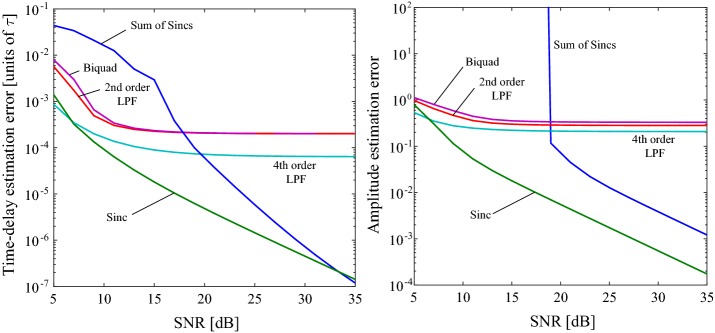
Time and amplitude estimation errors for various sampling kernels in the presence of noise for $L=4$ Dirac pulses ($F=4$)

### Architecture 2: quadrature synthetic aperture beamforming

The second architecture combines SAB with quadrature sampling—i.e. signals are processed in the baseband. The image is formed in the hardware front-end prior and then transmitted to the display device. The proposed quadratic SAB architecture is illustrated in Fig. 1b. The transducer produces a bandpass signal *R*(*t*) which may be expressed as:

10 $$\begin{aligned} R(t)&=A\left( t\right) \cos \left( \omega _{c}t+\phi \right) \end{aligned}$$

where $A\left( t\right)$ represents the envelope, $\omega _{c}$ the carrier frequency in radians per second, and $\phi$ the phase [16]. Expansion of *R*(*t*) yields:

11 $$\begin{aligned} R(t)&=A_{I}\left( t\right) \cos \left( \omega _{c}t+\phi \right) -A_{Q}\left( t\right) \sin \left( \omega _{c}t+\phi \right) \end{aligned}$$

where $A_{I}\left( t\right) =A\left( t\right) \cos \phi \left( t\right)$ and $A_{Q}\left( t\right) =A\left( t\right) \sin \phi \left( t\right)$ are the in-phase and quadrature components respectively. These may be obtained by mixing with a reference signal in the analogue domain and filtering the result. Since $A_{I}\left( t\right)$ and $A_{Q}\left( t\right)$ are baseband signals, they may be sampled at a lower rate. This reduces the computational burden on the beamforming processor (FPGA/ASIC). After sampling, the next step is to appropriately phase-rotate the I/Q data for focusing. According to the synthetic aperture focusing method, for a given pixel location $\overrightarrow{r_{p}}$ at depth index *k*, the required time instance $t_{p}(i,j)$ to take the signal value for summation is calculated by dividing this distance by the speed of sound in the medium [17].

12 $$\begin{aligned} t_{p}(i,j) &= \frac{|\overrightarrow{r_{p}}-\overrightarrow{r_{e}}(i)|+|\overrightarrow{r_{p}}-\overrightarrow{r_{r}}(j)|}{c} \end{aligned}$$

where $r_{p}$ denotes the position of the imaging point, $r_{e}(i)$ the location of the $i$th transmitting element and $r_{r}(j)$ the location of the jth receiving element. A corresponding discretised delay index $I_{p}(i,j)$ may then be calculated. An interpolation factor of *K* is applied to increase the delay resolution. That is, if $N_{S}$ sample points are obtained, then there are many as $K\times N_{S}$ index locations between 1 and $I_{p}(i,j)_{max}$. The index value is read from a lookup table that is calculated a priori, based on the locations of each pixel $(\overrightarrow{r_{p}})$ and transmitting (*i*) or receiving (*j*) element. For each index location $I_{p}(i,j)$, the I or Q data are then interpolated on-the-fly using any standard technique such as linear or quadratic interpolation.

Now, if the delay is applied directly to the I/Q data, critical frequency-dependent phase errors distort the final image [11]. Therefore, I/Q sample points are remodulated or upconverted back to RF by mixing the interpolated result with new discrete reference signals:

13 $$\begin{aligned} I_{ref}\left[ n\right]&=  \cos \left[ \omega _{c}n\right] \end{aligned}$$

14 $$\begin{aligned} Q_{ref}\left[ n\right] &= \sin \left[ \omega _{c}n\right] \end{aligned}$$

where $\omega _{c}$ is the carrier frequency in *rad*/*s* and *n* is the discretised time index. Again, $I_{ref}\left[ n\right]$ and $Q_{ref}\left[ n\right]$ are calculated a priori. The interpolated *I* and *Q* values are multiplied by the reference signals at $n=I_{p}$ and then subtracted to yield the RF amplitude:

15 $$\begin{aligned} R\left[ n\right]&= A_{I}\left[ n\right] \cos \left[ \omega _{c}n\right] -A_{Q}\left[ n\right] \sin \left[ \omega _{c}n\right] \end{aligned}$$

16 $$\begin{aligned}= A\left[ n\right] \cos \phi \left[ n\right] .\cos \left[ \omega _{c}n\right] -A\left[ n\right] \sin \phi \left[ n\right] \sin \left[ \omega _{c}n\right] \end{aligned}$$

17 $$\begin{aligned}= A\left[ n\right] \cos \left[ \omega _{c}n+\phi \right] \end{aligned}$$

This value is then added to the pixel location, and the process is repeated for all *i*, *j* and *n* values, resulting in a low-resolution image. These low-resolution images are summed or averaged to obtain a higher resolution image, which may then be transmitted via a wireless transmission link to an external post-processor. The final focused signal $y_{f}(\overrightarrow{r_{p}})$ expressed mathematically is:

18 $$\begin{aligned} y_{f}(\overrightarrow{r_{p}})= & {} \sum _{j=1}^{N}\sum _{i=1}^{M}a\left( I_{p}(i,j)\right) R\left( I_{p}(i,j)\right) \end{aligned}$$

where $a\left( I_{p}(i,j)\right)$ is the apodisation (weighting) function, $R\left( I_{p}(i,j)\right)$ is the phase-shifted I/Q sum evaluated at $I_{p}(i,j)$, *N* is the number of transducer elements and *M* the number of transmissions.

#### Design tradeoffs and optimisation

The parameters of the algorithm must be carefully selected to optimise the multidimensional tradeoffs between circuit area, power consumption, frame rate, image quality/size, transmission line bandwidth and system complexity/cost.

In particular, the image quality is dependent on the number of transmissions ($i_{max}$)/size of the synthetic transmit aperture, and the number of receivers ($j_{max}$)/size of the receive aperture. A larger value of $i_{max}$ implies better spatial compounding, SNR and lateral resolution. Similarly, the lateral resolution is a function of the size of the receive aperture, so increasing $j_{max}$ improves the image quality. However, increasing $i_{max}$/$j_{max}$ leads to an increase in data acquisition time and therefore a reduction in the *maximum* frame rate ($FR_{max}$), which is a function of the time of flight $t_{f}=2D/c$:

19 $$\begin{aligned} FR_{max}&=  \frac{N_{a}c}{2Di_{max}j_{max}} \end{aligned}$$

where *D* is the depth and *c* is the speed of sound in the medium. The maximum frame rate is linearly proportional to the number of parallel receiver channels $N_{a}$. $FR_{max}$ effectively defines the boundary of a region of operation for various values of $N_{a}$. For example, in Fig. 2, three regions of operation may be defined for $N_{a}=1$, $N_{a}=2$ and $N_{a}=8$. In this study, only a single channel is used as a proof of concept. Thus, with $N_{a}=1$, $D=10$ cm, $c=1540$ m/s, $i_{max}=30$ and $j_{max}=64$, the maximum frame rate is 4 Hz, which is acceptable for capsule endoscopy but not for a portable scanner. In this case, either the image size/quality may be reduced, or more channels must be used at the expense of increased power consumption.

The proposed algorithm inherently lends itself to an iterative, pipelined approach that may easily be implemented in a hardware description language (HDL) for implementation in hardware. Within the regions of operation discussed above, the frame rate is thus a function of other digital design parameters such as clock frequency, $f_{clk}$, and the degree of parallelisation (i.e. the number of parallel delay calculations per clock edge, $N_{p}$). In order to increase the frame rate up the maximum in (19), $f_{clk}$ and/or $N_{p}$ must be increased at the expense of power and/or area (or logic utilisation). For $N_{a}=1$, this relationship is expressed in the following equation:

20 $$\begin{aligned} FR&= \frac{N_{p}f_{clk}}{2 \cdot i_{max} \cdot j_{max}^{2} \cdot z_{max}} \end{aligned}$$

where $z_{max}$ is the number of pixels in the axial dimension of the image. Equation (20) may be derived using (18) by a process of multiplication. The beamforming process is carried out for all pixels ($j_{max}\times z_{max}$) for all transmit/receive operations ($i_{max} \times j_{max}$). A factor of two in the denominator is introduced to account for serialising send and receive operations in hardware over two clock cycles. The relationship in (20) is illustrated in Fig. 2, where frame rate is plotted against the number of transmit position, $i_{max}$, for various clock frequencies and $j_{max}=64$ channels, $z_{max}=350$, $N_{a}=1$ and $N_{p}=8$. Figure 2 also demonstrates the relationship between the clock frequency and $i_{max}$ for a constant frame rate of $5$ Hz. The clock frequency may be increased at the expense of power up to the maximum operating frequency of the digital circuit.

#### Dynamic apodisation

Dynamic apodisation is used to maintain a constant F-number ($f\#$) over the imaging depth. The F-number is defined as the ratio of the imaging depth, *z*, to the aperture size, $\alpha$ [18]. The synthetic aperture is dynamically grown as a function of the imaging depth in order to keep the $f\#$ constant. The number of lines *l* to consider in a window for focusing to a depth *z* are calculated using the following expression [18]:

21 $$\begin{aligned} l& {} = \frac{z_{k}}{(f\#)\cdot\triangle x} \end{aligned}$$

where $z_{k}$ is the pixel depth and Δ*x* is the inter-element spacing. This equation is used to derive a set of a priori constants that are stored in memory to allow for real-time dynamic apodisation.

### Experimental setup

In order to carry out measurements, a dedicated 2-layer printed circuit board (PCB) was designed, as shown in Fig. 3. The PCB interfaces with a full custom analogue front-end (AFE), which was designed and fabricated in AMS $0.35$ μm CMOS technology. The AFE functions as an analogue demodulator, producing I/Q components from an RF input signal. The chip includes a sixth order lowpass filter with selectable bandwidth to allow for testing over a range of values.

The PCB hosts a Cesys EFM-02 embedded FPGA module based on the Xilinx Spartan-6LX® FPGA (XC6SLX150-3FGG484I). The FPGA is used to generate control signals for the IC, as well as digital mixing signals. The FPGA also communicates with a ADC10D020 dual 10-bit ADC, which samples I and Q channels separately at 2.5 MHz. The quadrature SAB method and an internal UART module were implemented on FPGA. This module connects to an external FT232 USB to serial UART interface controlling communication with the PC. Image post-processing is carried out in MATLAB, which also handles PC-side serial communications.

A custom MATLAB program was written to control an external PicoScope® 5442B oscilloscope/arbitrary waveform generator (AWG). The script sequentially updates the AWG with RF signals obtained from a synthetic aperture database stored on the PC. This data were previously captured on a Verasonics Vantage 256™ system using a P4-1 phased array (central frequency at 2.5 MHz) with 96 active elements. The original RF sampling rate is 10 MHz, and the imaged medium was a wire phantom containing $8\times 3$ cross-sectional wires.

## Results and discussion

In this section, results for both architectures are presented. For the first architecture (FRI compressive SAB), simulations were initially carried out to validate the reconstruction accuracy of the algorithm. Thereafter, A-mode and B-mode imaging results were obtained using the hardware setup described above. B-mode images for the second architecture (quadrature SAB) were also obtained using the same hardware setup. Both architectures are compared in light of the resultant image quality and hardware efficiency.

### FRI compressive sensing results

#### Software simulations

The sampling scheme was first simulated using MATLAB in two scenarios prior to gathering hardware results. The scheme is demonstrated using ideal and noisy streams of Gaussian pulses in order to measure the accuracy of different FRI kernels. The MATLAB code used in these simulations is adapted from the code provided in [15, 19].

*Noiseless Case* Consider a noiseless input signal *x*(*t*) comprising $L=5$ delayed and weighted versions of a Gaussian pulse:

22 $$\begin{aligned} h(t)&=\frac{1}{\sqrt{2\pi \sigma ^{2}}}\mathrm{exp}\left( \frac{-t^{2}}{2\sigma ^{2}}\right) \end{aligned}$$

where $\sigma =3\times 10^{-3}$ and period $\tau =200$ μs. The time delays and amplitudes of all pulses in *x*(*t*) are allocated randomly. The signal is then convolved with the sampling kernel (lowpass filter, sinc, sum of sincs, etc). Assuming that an oversampling factor of $F=4$ is used, the low-rate sampling frequency is $f_{s}=\frac{N}{\tau }=\frac{2L\times F}{\tau }=\frac{2(4)(5)}{200\times 10^{-6}}=200$ kHz, which implies that the bandwidth of the filter must be $f_{c}=f_{s}/2=100$ kHz. When using an ideal sinc filter, the reconstructed signal is exact to numerical precision, as shown in Fig. 4a. However, when using a fourth order Butterworth lowpass filter, time delay errors are introduced between the input and reconstructed pulses due to the time constant of the filter (this may be corrected digitally after reconstruction). Amplitude errors are also presented, as shown in Fig. 4b. These errors may be minimised by increasing the order of the filter or by increasing the oversampling factor *F*, as discussed in the following section.

*Noisy case* Gaussian noise with variance $\sigma _{n}^{2}$ is added to the samples to test the performance of the sampling scheme in non-ideal conditions. The SNR is defined as [15]:

23 $$\begin{aligned} SNR&=\frac{\frac{1}{N}||c_{2}^{2}||}{\sigma _{n}^{2}} \end{aligned}$$

where *c* denotes the clean samples. For each SNR value in a range of $5-35$ dB, 400 experiments were carried out with unique noise vectors. The test signal was a series of $L=4$ Dirac pulses with an amplitude of unity. The time and amplitude errors are defined as the average of $||t-\hat{t}||_{2}^{2}$ and $||a-\hat{a}||_{2}^{2}$. Figure 5 illustrates the errors for various sampling kernels as a function of SNR. Since the causal filters introduce a time delay, the time error is calculated *after *shifting to correct the delay. The following filters are used in this analysis:

- Sinc filter: $s(t)=\mathrm{sinc}\left( Bt\right) ,$ where $B=1/T$.
- Sum of Sincs (SoS): ${s (t)\,=\,\mathrm{rect}\left( {\frac{t}{\tau }}\right) \sum _{k\in K}b_{k}e^{j2\pi kt/\tau }}$. In the frequency domain, ${S(s)=\frac{\tau }{\sqrt{2\pi }}\sum _{k\in K}b_{k}\mathrm{sinc}\left( \frac{\omega }{2\pi /\tau }-k\right) }$. The coefficients $b_{k}$ are set to 1, and $K=\left\{ -L,\ldots ,L\right\} .$
- Cascaded, first order lowpass filter: $S(s)=\frac{k}{(1+\frac{s}{\omega _{c}})}$. The gain $k=1$, and cutoff $\omega _{c}=2\pi \left( \frac{B}{2}\right) =\pi B$. First order stages are cascaded to form second or fourth order filters.
- Biquad filter: $S(s)=\frac{k\omega _{o}^{2}}{s^{2}+\frac{\omega _{o}}{Q}s+\omega _{o}^{2}}$, where $\omega _{o}$ is set to give the $-3$ dB cutoff $\omega _{c}$.

Evidently, the simple sinc kernel is more robust than the SoS kernel when the SNR is lower than 33 dB. For SNR values less than 19 dB, the response of the SoS kernel is unstable, whereas the responses of the other kernels are stable. For the causal filters (cascaded/biquad), the filter order is inversely proportional to the error, which tends towards a fixed value with increasing SNR. Causal filters naturally introduce systematic time delay errors. However, this may be corrected in software after reconstruction, as discussed in the previous section.

**Fig. 6:**
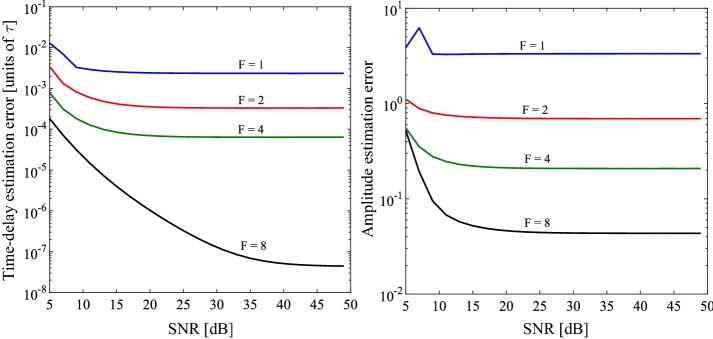
Time and amplitude estimation errors for oversampling factors of 1, 2, 4 and 8. In this case, a 2nd order LPF is used as the sampling kernel

**Fig. 7:**
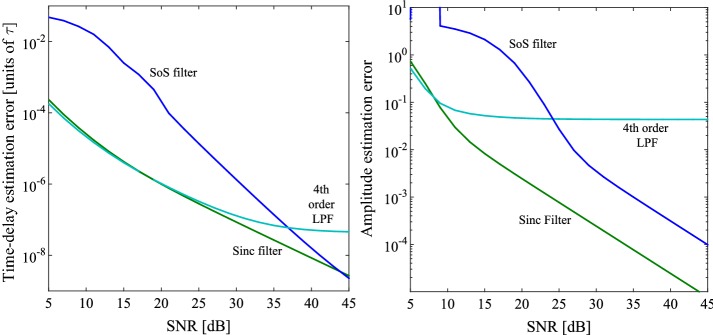
Time and amplitude errors for various sampling kernels when $L=4$, $F=8$. Note how the error for the 4th order cascade follows that of the sinc kernel for low SNR values

*Oversampling* The reconstruction accuracy may be improved by increasing the oversampling factor, *F*, at the expense of increased power consumption and transmission bandwidth. Figure 6 shows how the time and amplitude estimation errors change for different oversampling factors over a range of SNR values. In this case, a second order lowpass filter was used as the sampling kernel, and 500 experiments were carried out for $L=4$ over a range of oversampling factors (1, 2, 4 and 8). Clearly, the estimation errors decrease as the oversampling factor increases. As the SNR increases beyond 40 dB, the time error decreases from $6.4\times 10^{-5}$ τ ($F=4$) to $4.5\times 10^{-8}$ τ ($F=8$) and the amplitude error from 0.208 ($20.8\%$) to 0.04 ($4\%$). For low SNR values, the estimation errors tend toward those of the sinc kernel. This trend is illustrated in Fig. 7, which highlights the performance of the sampling kernels when *F* is increased to 8. An optimal response may be approximated by either increasing *F* or the order of the filter. However, in doing so, the inherent tradeoffs must be carefully considered.

**Fig. 8:**
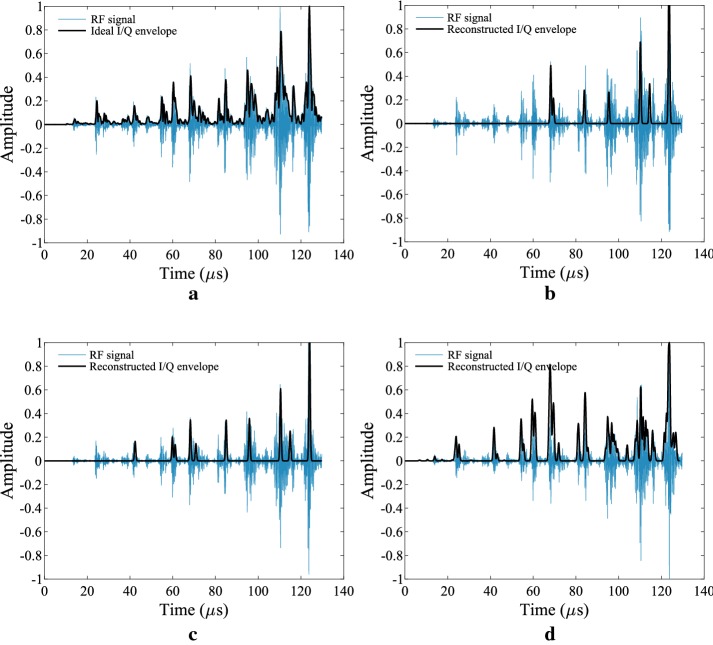
Original versus reconstructed envelopes. In ** a**, the original RF signal is overlayed against the ideal I/Q envelop generated in software. Low-rate samples are obtained using the hardware front-end and the I/Q envelop is reconstructed using FRI CS with the following parameters: ** b** $L=7.$ **c** $L=17.$** d** $L=60$

**Fig. 9:**
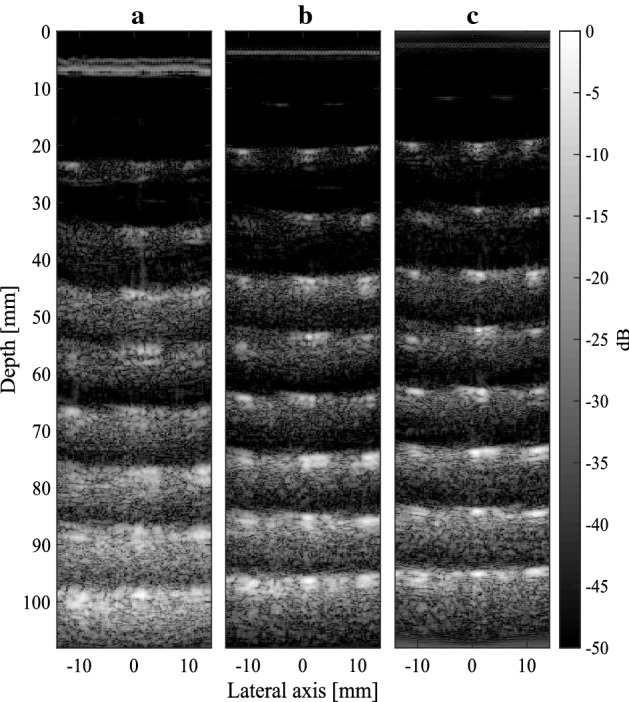
Images of a phantom containing $8\times 3$ cross-sectional wires. Compressive SAB was carried out with 48 transmit elements ($f\#=2.5$), and **a** $L=7,$ **b** $L=17$ and **c** $L=40$

**Fig. 10:**
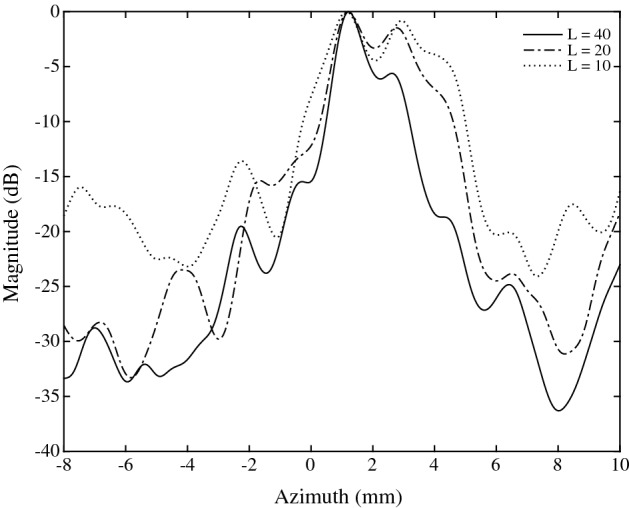
Lateral beamplots ($i_{max}=48$, $f\#=2.5$, $z=66.5$ mm) demonstrating the effect of *L* on the lateral resolution

#### Hardware results

*A-line Envelop Reconstruction* In order to validate the functionality of the compressive sensing algorithm in hardware (prior to beamforming), experiments were carried out using a single A-mode signal derived from the database described in the Methods section. The A-line signal may be modelled as a 1D stream of Gaussian pulses with width $\sigma =3\times 10^{-7}$. After demodulating and filtering below the Nyquist frequency, each I/Q signal is sampled at frequency $f_{s}$ and then reconstructed using the method in [13]. The results of the experiment are shown in Fig. 8. The number of samples per time window $\tau$ is $N=2L$, where *L* is the number of Gaussian pulses per period. Three experiments were carried out for each cutoff frequency in the AFE. The parameters for these experiments are defined in Table 1. Figure 8b–d show the results for experiments 1, 2 and 3 respectively. The ideal envelop is shown in Fig. 8a. By observation, larger values of *L* yield better reconstruction accuracies, with the tradeoff being an increased sampling rate.

**Table 1:** Parameters and image quality measurements for the FRI compressive SAB method

	Experiment 1	Experiment 2	Experiment 3	RF Ref
$f_{s}$	390 kHz	1.02 MHz	3.7 MHz	10 MHz
$f_{c}$	195 kHz	510 kHz	1.85 MHz	5 MHz
*L*	7	17	40	N/A
$SNR$ (dB)	23	27	34	42
$LR$ (mm)	4.49	3.13	2.07	1.2
$RC$ (dB)	22	31	35	43.5

Image quality is quantified using *SNR*, lateral resolution (*LR*) and relative contrast (*RC*)

**Table 2:** Device utilisation summary on a Spartan-6 FPGA for $N_{p}=16$, frame rate = 7 Hz, pixel resolution = $64\times 352$ and $i_{max} = 16 \,{\rm angles}$

Logic utilisation	Units	Device utilisation (%)
Slice Registers	7576	4
Slice Look-up tables (LUTs)	28,017	30
LUT-FF pairs	1926	3
Block RAM/FIFO	32	11
DSP48A1s	4	0.1
Global Buffers (BUFG/BUFGCTRL)	1	25

**Table 3:** Parameters and image quality measurements for the quadrature SAB method

	Experiment 1	Experiment 2	Experiment 3	RF Ref
$i_{max}$	3	16	48	48
$SNR$ (dB)	29.1	33.6	38.2	42
$LR$ (mm)	2.5	1.25	1.2	1.2
$RC$ (dB)	30.2	35	39.5	43.5

Image quality is quantified using *SNR*, lateral resolution (*LR*) and relative contrast (*RC*). $i_{max}$ is the number of transmission positions

**Table 4:** Performance comparison for various beamforming architectures

Paper	Technology	Channels	Beamformer architecture	Center freq (MHz)	Delay resolution (ns)	Frame rate	Power/channel (mW)
This work	Spartan-6™ FPGA	64 (1 Rx channel)	Quadrature, single channel, digital SAB	$2.5$	$100$	$7$ Hz	$4.1$
[20]	$0.18$ μm CMOS	$32\times 32$	Analogue, sample-and-hold sub-array beamformer	$5$	$30$	$44.4$ vol/s	$0.27$
[9]	$0.13$ μm NAND	32	Parallel digital delay-and-sum	$3.5$	$17.8$	$30$ Hz	$9.5$
[6]	$0.13$ μm CMOS	32	Analogue, sample-and-hold sub-array beamformer	$1.25$	1.75–$2.5$	–	$8.9$
[5, 21]	$0.35$ μm CMOS	8	Parallel delay-and-sum using analogue delay cells	30–50	1.75–$2.5$	–	$8.4$
[22]	Spartan-3 FPGA	32 (16 Rx channels)	Pseudo-dynamic, extended aperture, digital beamformer	$3.5$	–	$30$ Hz	–

The original RF sampling frequency is 10 MHz. Thus, the sampling rate (for both I and Q) is reduced by a factor of 12.8, 4.9 and 1.4 for each experiment respectively. Thus, for $L=60$, there is no advantage in using compressive sensing as the sampling rate is higher than the ideal I/Q Nyquist sampling rate. There is thus a tradeoff between *L*, the reconstruction accuracy and the sampling rate. To achieve higher accuracy, both *L* and *F* should be large, but this results in an increased sampling rate and power consumption. The circuit topology realising the CS framework should be tuneable to maximise the performance and minimise the sampling rate.

*B-Mode Imaging* Finally, the compressive SAB architecture was evaluated by producing a full B-mode image using the RF dataset. Raw RF signals were sequentially demodulated in hardware using the cutoff frequencies in experiment 1 and 2, corresponding to *L* values of 7 and 17. Subsequent beamforming was carried out in MATLAB using the synthetic aperture method for the following parameters: $j_{max}=64$, $k_{max}=352$ (i.e. pixel resolution of $64\times 352$), $i_{max}=48$. The resultant images are shown in Fig. 9a ($L=7$), 9b ($L=17$), and 9b ($L=40$). These images may be compared against the “ideal” RF-beamformed reference in Fig. 12c. Lateral beamplots are also presented in Fig. 10, demonstrating the effect of *L* on lateral resolution. These beamplots were taken from a point target located at a depth of 66.5 mm, and correspond to a grayscale magnitude in decibels. The lateral resolution, SNR and contrast are calculated for each *L* value and presented in Table 1. Lateral resolution (LR) is defined as the full width at half maximum (FWHM), i.e. $-\,6$ dB main lobe width. Relative contrast (RC) is defined as the ratio of the maximum grayscale magnitude at the point target to the average magnitude of the background. The SNR is the ratio of the mean to standard deviation of the image.

For $L=7$, the SNR and lateral resolution is poor since fewer Gaussian pulses are used to reconstruct the I/Q signals. Increasing the number of Gaussian pulses *L* increases the reconstruction accuracy and thus improved image quality. In particular, for $L=40$, the SNR is 34 dB, and lateral resolution (2.07 mm) is closer to the ideal reference (1.2 mm). However, increasing *L* eventually pushes the low-rate sampling above that of the Nyquist quadrature sampling frequency.

The normalised root-mean-square-error (NRMSE) may be used as a quantitative measure of image quality for “ideal” RF-domain beamforming and FRI compressive beamforming. The NRMSE is computed on a scan-line/columnwise basis by comparing each pixel in the RF reference image, $g_{j,k}$, to that of the measured image, $f_{j,k}$, as follows:

24 $$\begin{aligned} NRMSE= \frac{1}{K}\sum _{k=1}^{K}\frac{\sqrt{\frac{1}{J}\sum _{j=1}^{J}\left( f_{j,k}-g_{j,k}\right) ^{2}}}{max\left( g_{j,k}\right) -min\left( g_{j,k}\right) } \end{aligned}$$

where $max\left( g_{j,k}\right)$ and $min\left( g_{j,k}\right)$ represent the maximum and minimum values of each column in $g_{j,k}$ respectively. For $L=7$ and $L=17$, the NRMSE is 26 and $22\%$ respectively, in comparison with the “ideal” RF case.

### Quadrature SAB results

**Fig. 11:**
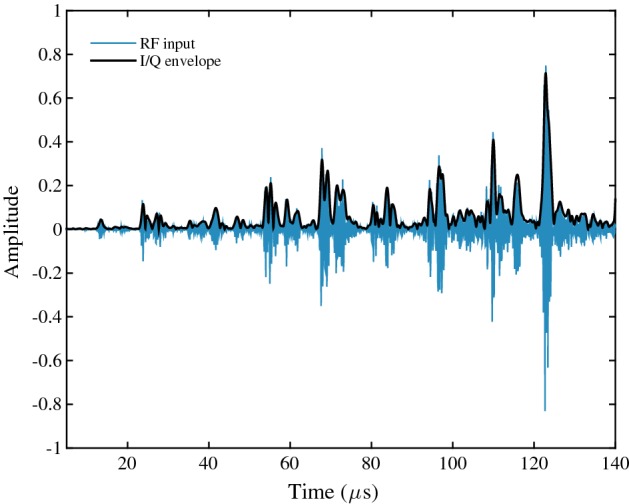
RF and envelop signals obtained using analogue front-end The RF signal was demodulated by the AFE, yielding I/Q signals, where were used to calculate the envelop

A-mode results were obtained by demodulating RF signals using the analogue front-end. Note that the lowpass filter cutoff frequency was set equal to the baseband signal bandwidth (1.25 MHz). The individual I/Q sampling rate is 2.5 MHz, since the I/Q baseband bandwidth is 1.25 MHz. Thus, the combined I/Q sampling rate (5 MHz) is reduced by a factor of 2 from the original 10 MHz RF sampling rate. The resultant I/Q signals were used to calculate the A-mode envelop, which is displayed in Fig. 11. The quadrature SAB method was tested in a similar manner by producing a B-mode image using the same RF dataset. The difference in this case is that demodulated signals from the analogue front-end were processed by the digital quadrature beamformer implemented using a Spartan-6LX® FPGA (XC6SLX150-3FGG484I). The device utilisation summary is provided in Table 2 for the following parameters: $f_{clk}=20$ MHz, $\mathrm{frame rate}=7$ Hz, $N_{p}=16$, $i_{max}=48$, $j_{max}=64$, $k_{max}=352$ (i.e. pixel resolution of $64\times 352$). On-chip BRAM was used to store the image and beamforming/apodisation parameters.

The on-chip power consumption is proportional to the system clock frequency ($f_{clk}$). For $f_{clk}=20$ MHz, the power consumption is estimated to be $296$ mW (static power 172 mW, dynamic power $124$ mW) by the Xilinx power estimator. This works out to be an equivalent power consumption of 4.6 mW/channel across the entire synthetic aperture (64 elements). Doubling the clock frequency allows for better spatial compounding (larger $i_{max}$), as well as a higher frame rate or pixel resolution, at the expense of doubled power consumption and half the battery life.

**Fig. 12:**
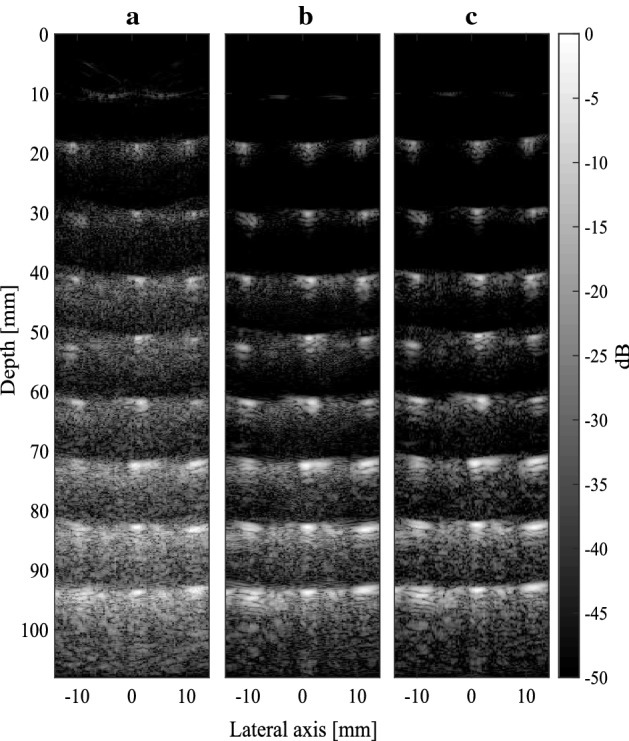
Images of a phantom containing $8\times 3$ cross-sectional wires. In** a**,** b**, quadrature beamforming is carried out with 16 and 48 transmit elements respectively ($f\#=2.5$). In **c** beamforming is carried out in the RF domain with 48 elements

**Fig. 13:**
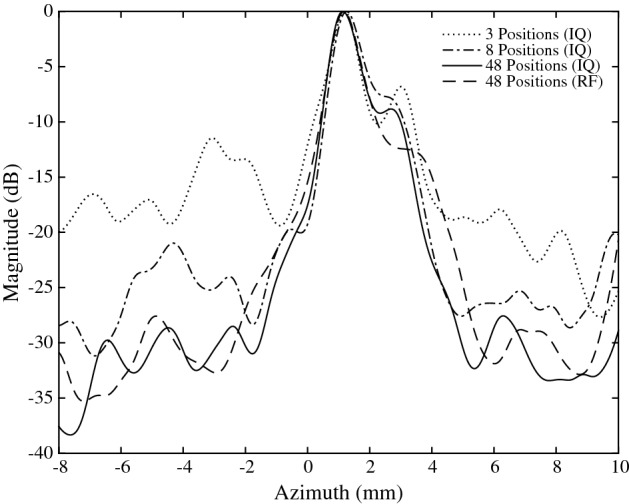
Lateral beamplots for 3, 16 and 48 transmitter positions ($f\#=2.5$, $z=66.5$ mm)

Three experiments were carried out for different values of $i_{max}$. The results are provided in Table 3. Note that SNR, lateral resolution and relative contrast are calculated using the same method as in the compressive SAB section. Figure 12 compares the resultant B-mode images for $i_{max}=16$ and $i_{max}=48$ against the “ideal” RF beamforming reference (Fig. 12c). For $i_{max}=48$ (Fig. 12b), the NRMSE is $12.5\%$ and the lateral ($1.2$ mm) is identical to the RF case. When decreasing the number of transmissions to 8, the NRMSE increases to $16.5\%$ due to a reduction in the SNR caused by larger sidelobes and increased speckle noise. The reduction in image quality is qualitatively evident in Fig. 12a and in the lateral beamplots in Fig. 13, where the greyscale magnitude is plotted against lateral width. Decreasing $i_{max}$ from 48 to 3 results in a 9.1 dB SNR reduction. Lateral resolution increases from 1.2 to 2.5 mm. However, according to equation (20), there is an inverse relationship between the $i_{max}$ and frame rate. Decreasing $i_{max}$ from 48 to 8 elements leads to an increase in frame rate from 2.5 to 15 Hz. Similarly, for a constant frame rate, a sixfold reduction in the number of transmissions leads to a proportional decrease in power consumption, since the system clock frequency may be decreased or the area/logic capacity reduced as fewer pixels are calculated in parallel.

**Fig. 14:**
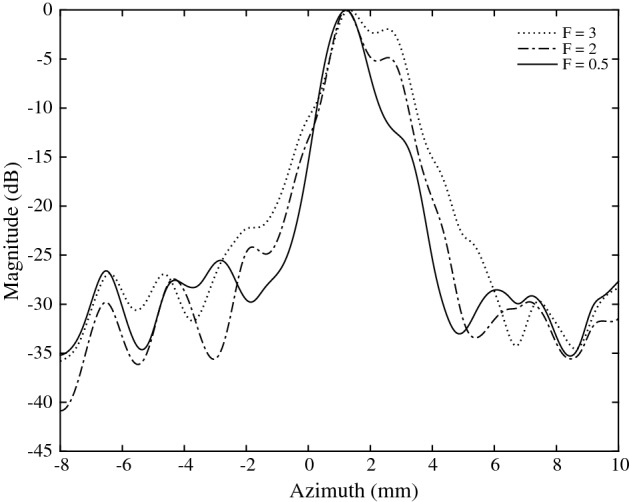
Lateral beamplots for $f\#=0.5$, 2 and 3 ($z=66.5$ mm, $i_{max}=48$)

**Fig. 15:**
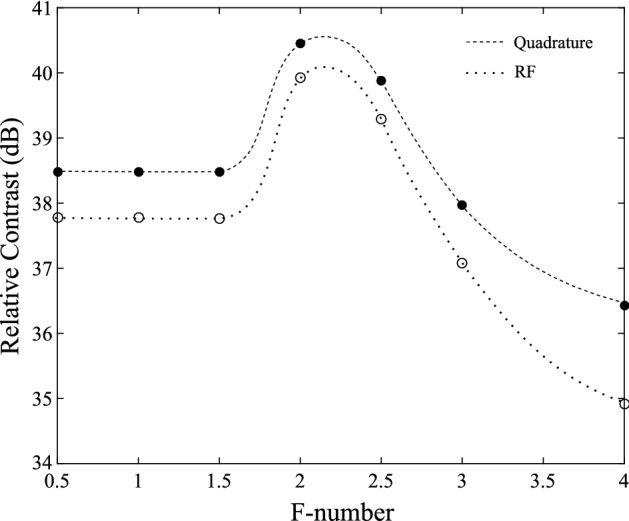
Contrast relative to the average background value for various $f\#$ values ($z=66.5$ mm, $i_{max}=48$)

The $f\#$ is also an important parameter affecting the width of the main lobe, and thus the lateral resolution. In Fig. 14, it can be seen that the main lobe width increases as $f\#$ increases from 0.5 to 3. Thus, better focusing is achieved with a smaller $f\#$. The $f\#$ also affects the relative contrast, as shown in Fig. 15. For RF and quadrature beamforming, the contrast increases to a maximum of 40 and 40.5 dB at $f\#=2.2$, after which it gradually decreases.

### Comparison of architectures

Both architectures employ an identical hardware front-end, which demodulates RF ultrasound signals prior to beamforming. In the quadrature method, beamforming is carried out by an FPGA in the hardware front-end, yielding a B-mode image which may then be transmitted to the back-end for display. The combined I/Q sampling rate (5 MHz) is half that of the original RF. At an RF sampling frequency of 10 MHz, the ADC power consumption is 75 mW. A reduction in sampling rate translates to a linear reduction in ADC power consumption (37.5 mW for both I/Q channels sampled at 2.5 MHz). Additionally, a proportional decrease in memory capacity (with fewer samples) leads to a reduction in logic area and power consumption. The system power (excluding that of the transmission link) may be estimated by summing the power of the AFE (7.8 mW), digital beamformer (269 mW) and ADC (37.5 mW)—i.e., 314.3 mW. On the other hand, the compressive SAB architecture merely bandlimits signals in the analogue domain, and transmits low-rate samples to a computational back-end for image reconstruction. This even further reduces the sampling rate and data bandwidth. In this case, the power consumption (excluding that of the transmission link) is the sum of the AFE (7.8 mW), and ADC (75 mW for $L=17$). Thus, the total power is lower, but the tradeoff is image quality quality.

The lateral resolution for the quadrature SAB case (1.2 mm) is identical to the RF reference for the same number of transmissions. For the compressive SAB method, lateral resolution is significantly poorer (2.07 mm) for the best case ($L=40$). For this *L* value, SNR and relative contrast are similar to the quadrature SAB method. However, at lower *L* values, image quality becomes unacceptably poor due to sparse signal reconstruction of the I/Q envelop. Thus, it is evident that the quadrature SAB method yields better image quality (for an identical number of transmissions) than the compressive SAB method. The NRMSE for the quadrature architecture was $9\%$ lower for the same $i_{max}$ value (48). *L* should be increased beyond 17 in order to achieve image quality that is comparable to the quadrature SAB case. This in turn increases the bit-rate and power consumption of the transmission link. For instance, for $L=17$ ($f_{s}=1.05$ MHz), the required bit rate is $2\times 1.05\times 10=21$ Mbps, which is 4.7 times lower than the bit rate required to transmit RF samples at 100 Mbps. Transmission at this frequency is feasible using a typical 2.4 GHz, 802.11 g transceiver, for example, which operates up to a maximum of 54 Mbps. The frame rate is constrained, however, and more than one channel cannot practically be used due to the limited transmission line bandwidth. However, in the case of the quadrature method, the frame rate may be increased by adding more multiple channels at the expense of increased power consumption.

Table 4 compares various state-of-the-art beamforming architectures with the proposed quadrature SAB architecture. The key advantage of the proposed architecture is a reduced number of analogue receiver channels, leading to reduced system complexity, area and cost on a system-level. The digital beamformer power consumption per channel (4.1 mW) is comparable to state-of-the-art digital and mixed-signal beamformers, on a per-channel basis. However, a proper comparison of the proposed digital receive beamformer with prior art is challenging as other designs focus on different imaging applications and have varying numbers of channels. For instance, the state-of-the-art mixed-signal beamformer in [20] consumes $276$ mW across all $32\times 32$ channels, which is slightly more than the proposed design ($262\,{\rm mW}$ across 64 channels). However, while the per-channel power in [20] is lower, scan-line conversion is not carried out on-chip, so offloading scan-lines at wireless transmission rates would be a significant challenge. On the other hand, the proposed beamformer converts I/Q signals to a B-mode image as a means of compressing data prior to conversion. This is because the proposed design targets small-scale wireless applications.

The natural tradeoff for both architectures is frame rate—for $i_{max}=8$, the frame rate (15 Hz) is half that of prior art. For a single analogue channel ($N_{a}=1$), the maximum frame rate is limited by the reflection or acquisition time. The frame rate can be increased for the same $i_{max}$ if the number of parallel analogue channels ($N_{a}$) is increased to 2 or more at the expense of increased power consumption. The delay resolution is also lower than that of prior art due to a relatively low oversampling factor. Future work will involve increasing the delay resolution by increasing the interpolation factor.

## Conclusion

Two architectural solutions are compared for highly miniaturised ultrasound imaging applications. The architectures combine synthetic aperture beamforming (SAB) with quadrature sampling and compressive sensing respectively, in order to reduce the power consumption, cost, circuit area and the complexity of the receiver. The sampling rate is reduced by a factor of 2 (quadrature SAB) and 4.7 (compressive SAB), compared to the RF sampling rate. The quadrature SAB method yields a higher image SNR and $9\%$ lower root mean squared error with respect to the RF-beamformed reference image than the compressive SAB method. The quadrature method is implemented on FPGA, with a total power consumption of 4.1 mW. Both architectures achieve a significant reduction in sampling rate, system complexity and area, allowing for aggressive miniaturisation of the imaging front-end.
